# Extending the Indications for Primary Nerve Surgery in Obstetrical Brachial Plexus Palsy

**DOI:** 10.1155/2014/627067

**Published:** 2014-01-12

**Authors:** Stuart A. Bade, Jenny C. Lin, Christine G. Curtis, Howard M. Clarke

**Affiliations:** ^1^Department of Plastic and Reconstructive Surgery, Hospital for Sick Children, Toronto, ON, Canada M5G 1X8; ^2^Royal Children's Hospital, Herston Road, Herston, Brisbane, QLD 4029, Australia; ^3^University of Montreal, Montreal, QC, Canada H3T 1C5; ^4^Department of Surgery, University of Toronto, Toronto, ON, Canada M5T 1P5

## Abstract

*Purpose.* This study identifies a small subset of patients with obstetrical brachial plexus palsy who, while they do not meet common surgical indications, may still benefit from primary nerve surgery. *Methods.* Between April 2004 and April 2009, 17 patients were offered primary nerve surgery despite not meeting the standard surgical indications of the authors. The authors performed a retrospective analysis of these 17 patients using prospectively collected data. *Results.* This group of 17 patients were identified as having poor shoulder function at about 9 months of age despite passing the Cookie Test. Fourteen patients underwent surgical intervention and three families declined surgery. All patients in the operative group regained some active external rotation after surgery. Five patients in this group have required further interventions. Two of the three patients for whom surgery was declined have had no subsequent spontaneous improvement in active external rotation. *Discussion.* The commonly used indications for primary nerve surgery in obstetrical brachial plexus palsy may not adequately identify all patients who may benefit from surgical intervention. Patients who pass the Cookie Test but have poor spontaneous recovery of active shoulder movements, particularly external rotation, may still benefit from primary nerve surgery.

## 1. Introduction

The incidence of obstetrical brachial plexus palsy is between 0.5 and 3 injuries per 1000 live births [1–5]. The majority improve spontaneously without surgical intervention [1]. For those patients who do not make a satisfactory spontaneous recovery, there are a variety of opinions regarding surgical indications for which there is no consensus. Gilbert has popularized the most commonly used indication for operative management in obstetrical brachial plexus palsy [6–8]. He advocated neuroma excision and interpositional nerve grafting in patients where there was no recovery of biceps function at 3 months of age. However it has been shown that there is a subset of patients who have biceps function at 3 months but may benefit from surgery [9]. Also there are patients who have no biceps function at 3 months and may make a satisfactory recovery without surgical intervention [1, 9].

**Figure 1:**
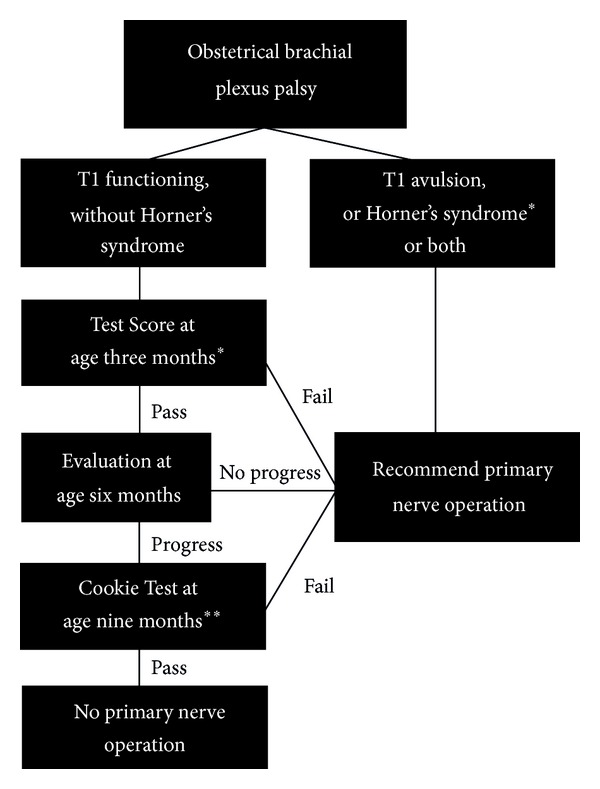
Flow diagram depicting the standard indications for primary nerve surgery in our clinic. *Test based on statistically analyzed data. **Test based on published empirical evidence (Reprinted with permission from [11]).

Our indications for primary surgery have been reported in detail in previous publications (Figure 1) [10–12]. We use the active movement scale (AMS) to grade fifteen upper limb movements (Table 1). At 3 months of age, a Test Score is determined using the active movement scale scores for elbow flexion, elbow extension, wrist extension, thumb extension, and finger extension. If the Test Score is less than 3.5 at 3 months of age, or if there is evidence of T1 nerve root avulsion or a Horner's syndrome, then surgical management is recommended. For those that do not meet these criteria, the final decision regarding surgical management is deferred until a later visit. At 9 months of age a Cookie Test is performed [10]. If the child fails the Cookie Test operative management is recommended. In cases selected based on the surgeons' experience surgery may be recommended at 6 months of age if there has been little or no improvement since the 3-month visit and the probability of passing the Cookie Test if recovery was allowed until 9 months of age is deemed to be small [11].

We feel, however, that there are some patients who do not meet any of these indications and yet may benefit from primary nerve surgery. Despite having a 3-month Test Score greater than 3.5 and despite passing the Cookie Test at 9 months of age, there is a small subset of patients who demonstrate limited recovery of the shoulder, particularly external rotation, who may be improved by a primary nerve operation.

This is a preliminary paper to outline our extended indications for primary nerve surgery in obstetrical brachial plexus palsy and to describe the results in the patients who have met these indications.

## 2. Materials and Methods

Between April 2004 and April 2009, 291 new infants less than one year of age were seen in the multidisciplinary Brachial Plexus Clinic at our institution. Of these, 41 patients met our standard indications for primary surgery (Figure 1) and went on to have exploration of the brachial plexus with excision of the neuroma and reconstruction with nerve grafts and/or nerve transfers. During the same period, we identified a consecutive subset of all patients who, despite having a 3-month Test Score greater than 3.5 and despite passing the Cookie Test at 9 months, had sufficient passive range of motion of their shoulder to reveal limitations in their active shoulder movement (absent external rotation with or without limited shoulder flexion and abduction). These 17 patients were offered surgery to address poor spontaneous recovery of shoulder function. A retrospective analysis of these 17 patients was performed using prospectively collected data. This study was approved by the Research Ethics Board at our institution.

A prehoc power analysis [13, 14] was undertaken to determine the sample size required to disprove the null hypothesis that the results of surgery were identical to those of nonoperated patients. At a significance level of 0.05 (alpha) and a power of 0.81 and allowing for a difference between groups of one grade and a standard deviation of 1, a minimum of 17 patients would be required in each of the operated and nonoperated groups to disprove the null hypothesis. Given that we had currently assembled only 3 patients in the non-operated group, we elected to proceed with a descriptive report since obtaining sufficient data for statistical analysis would require either decades of further data collection or a prospective randomized trial.

## 3. Results

Seventeen patients were identified as having poor shoulder function at about 9 months of age despite passing the Cookie Test. None of these patients had prior surgery or botulinum toxin injections. Sixteen of these patients had no active external rotation (AMS = 0). The remaining patient had a preoperative external rotation AMS of 2, as well as scores of 2 for shoulder flexion and abduction. All patients had sufficient passive range of motion to reveal limitations in their active shoulder movements. Three families declined surgery. Fourteen patients underwent surgical intervention at an average age of 10.3 months (range 8.6–13.2 months) (Table 2).

Nerve transfer of the distal accessory nerve to the suprascapular nerve was performed as an isolated procedure in 7 patients. Four patients had accessory to suprascapular nerve transfer performed along with botulinum toxin injections into the internal rotators at the time of surgery. The three remaining patients underwent exploration of the brachial plexus, neuroma excision, and reconstruction of the upper trunk or upper and middle trunks by sural nerve grafting with or without accessory nerve transfer. These latter three patients, as well as having poor external rotation, also had poor shoulder flexion and abduction warranting a full reconstruction of the injured brachial plexus.

One patient has had less than 6-month follow-up and is excluded from the analysis. The remaining 13 operative patients have been followed up for between 6 and 58 months (mean 23 months). All patients gained some active external rotation at some point in time postoperatively, ranging from 2 to 7 on the active movement scale. All patients have regained or surpassed their preoperative scores for other shoulder movements. However three patients have required botulinum toxin injections into the internal rotators postoperatively. One of these patients has subsequently proceeded to secondary shoulder reconstruction with a subscapularis slide, latissimus dorsi and teres major tendon transfers, and glenoid osteotomy performed for poor active external rotation, a posteriorly subluxed shoulder and marked glenoid dysplasia. Secondary shoulder reconstruction has been recommended to one additional patient but has not yet been performed. One patient who initially was scored 2 for external rotation at 3 months after surgery now has no observable active external rotation at 6 months after surgery; it appears that the internal rotators have overpowered the reinnervating external rotators and botulinum toxin injections have been recommended.

Of the three patients who refused surgery, one patient has recovered some external rotation spontaneously, scoring 2 on the active movement scale at the latest follow-up. The remaining two patients have not recovered any active external rotation on subsequent follow-up (Table 3).

## 4. Discussion

Early operative management, consisting of nerve grafts and nerve transfers, has been accepted as the standard of care for total plexus injuries [11]. However there is no consensus regarding the indications for and timing of surgical management for subtotal obstetrical brachial plexus injuries.

Gilbert et al. [6–8] have popularised the most commonly used indication for primary nerve surgery in obstetrical brachial plexus injuries. They advocate neuroma excision and interpositional nerve grafting in cases where no biceps function is present at 3 months of age. This is based on the finding that at 5 years of age shoulder outcomes were poor in those children that failed to spontaneously develop elbow flexion by 3 months of age. However, Michelow et al. [1] determined that elbow flexion alone at 3 months of age incorrectly predicted a poor recovery 12 percent of the time. Michelow et al. showed that the accuracy of predicting outcome improves when elbow flexion at 3 months is combined with elbow, wrist, finger, and thumb extension into a Test Score. Fisher et al. [9] confirmed that the presence of early elbow flexion alone is not sufficient to recommend a nonoperative approach. This study also showed that there is a small subset of patients with absent elbow flexion at 3 months of age who go on to have a spontaneous recovery of useful upper extremity function. Waters determined that surgical management for lack of biceps recovery at 6 months of age produced better outcomes than those children who were managed nonoperatively and had spontaneous recovery of biceps function by 5 months. Therefore he recommended surgery for those children who have not spontaneously recovered biceps function by 5 months of age and those with flail upper extremities with Horner syndrome [15, 16].

Isolated accessory to suprascapular nerve transfer has been previously reported to improve poor external rotation in the setting of otherwise satisfactorily recovered obstetrical brachial plexus lesions [17, 18]. However, these studies present either limited follow-up or different surgical indications from our study.

**Figure 2:**
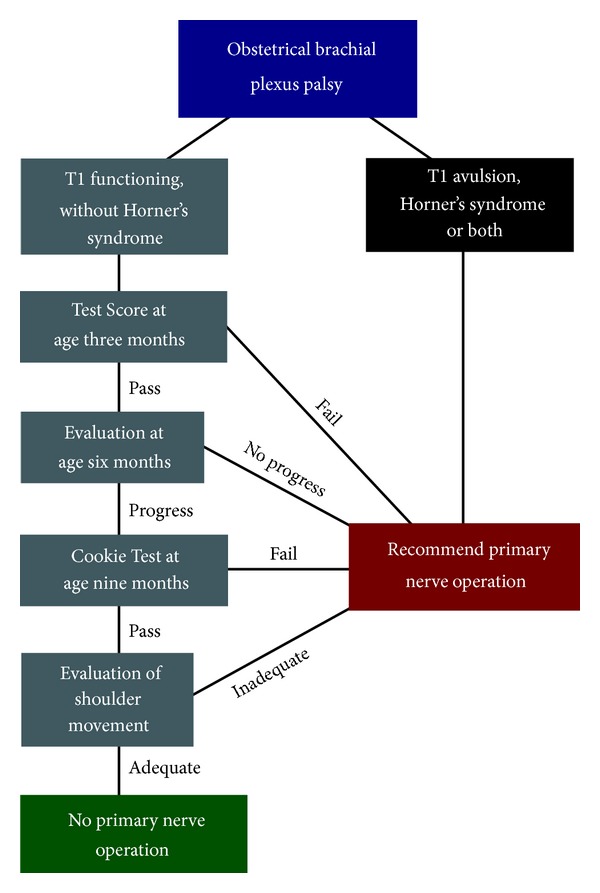
Flow diagram depicting the extended indications for primary nerve surgery in our clinic.

Obstetrical brachial plexus lesions are complex injuries with a wide range of severity and prognosis. The patterns of reinnervation and recovery are neither completely understood nor completely predictable. We believe that it is impossible to prognosticate accurately in every child at a single age with a single examination. This has led to the development of the Test Score at 3 months and the Cookie Test at 9 months of age. The standard indications for primary nerve surgery that are used in our multidisciplinary clinic have been described in detail in previous publications and are outlined in Figure 1 [10–12]. More recently we have further expanded the indications for primary surgery as presented in this paper (Figure 2). Patients who pass the Cookie Test but have poor active shoulder function (particularly external rotation) and good passive range of motion may benefit from surgery. Isolated accessory to suprascapular nerve transfer or neuroma excision and reconstruction are surgical options in this treatment group. Botulinum toxin injections may be a useful adjunct to surgical reconstruction in some patients. Achieving AMS grade 2 external rotation of the shoulder in these patients may be sufficient to provide adequate function.

It is impossible to make substantive conclusions in this study based on a small number of patients, limited follow-up, and the absence of a control group. Ideally long-term outcomes including functional assessments and rates of secondary shoulder surgery will be studied. However this will always be a small, carefully selected subset of patients. In this series all patients gained some measure of external rotation at some point in time in the postoperative period, although this did not necessarily obviate the need for further interventions—two patients in this study have been recommended to have secondary shoulder surgery and 3 additional patients have had subsequent botulinum toxin injections.

Importantly, no family reported a reduction in patient function due to the surgery. As has previously been shown [19], there is no long-term functional morbidity from harvesting the sural nerve for nerve grafting in children. In addition, we have not been able to identify any measurable difference in power or function of the trapezius muscle after accessory nerve transfer in our experience of over 150 cases. Given the low risk of morbidity and the potential functional gains achieved in some patients, primary nerve surgery can be considered in those patients that meet the extended indications described in this paper (Figure 2).

## 5. Conclusion

The commonly used indications for primary nerve surgery in obstetrical brachial plexus palsy may not adequately identify all patients who may benefit from surgical intervention. In this paper we have suggested a further subset of patients who may benefit from surgery. There are a small number of patients who make a satisfactory spontaneous recovery except for poor shoulder function, particularly external rotation. Consideration can be given to extending the indications for primary nerve surgery to include these patients, in whom satisfactory shoulder movement may be achieved with a primary nerve operation alone. Investigation is ongoing to further define the indications and outcomes in this subset of patients.

## Figures and Tables

**Table 1:** The Hospital for Sick Children Active Movement Scale*.

Observation	Muscle grade
Gravity eliminated	
No contraction	0
Contraction, no motion	1
Motion ≤ 1/2 range	2
Motion > 1/2 range	3
Full motion	4
Against gravity	
Motion ≤ 1/2 range	5
Motion > 1/2 range	6
Full motion	7

*Reprinted with permission from [10].

**Table 2:** Results: patients who underwent surgery.

	Age at surgery (months)	Surgery details	Preoperative AMS for external rotation	Postoperative AMS for external rotation	Length of Follow-up (months)
1	9.6	Isolated accessory to suprascapular nerve transfer	0	3	57.9
2	10.6	Isolated accessory to suprascapular nerve transfer	0	7	34.2
3	10.1	Isolated accessory to suprascapular nerve transfer	0	7	26.7
4	11.0	Sural nerve graft from C5 and C6 to suprascapular nerve and upper trunk	0	2	42.8
5	9.8	Accessory to suprascapular nerve transfer and botulinum toxin injections to shoulder internal rotators	0	2	34.8
6	11.5	Isolated accessory to suprascapular nerve transfer	0	3	9.4
7	10.4	Isolated accessory to suprascapular nerve transfer	0	2	25.9
8	13.2	Accessory to suprascapular nerve transfer and botulinum toxin injections to shoulder internal rotators	0	2	22.3
9	10.4	Sural nerve graft from C5 and C6 to upper trunk and accessory to suprascapular nerve transfer	0	2	21.6
10	9.6	Isolated accessory to suprascapular nerve transfer	0	3	12.4
11	9.3	Sural nerve graft from C5, C6, and C7 to upper and middle trunks and accessory to suprascapular nerve transfer	2	1	14.4
12	9.1	Isolated accessory to suprascapular nerve transfer	0	7	12.2
13	12.0	Accessory to suprascapular nerve transfer and botulinum toxin injections to shoulder internal rotators	0	0	6
14*	8.6	Accessory to suprascapular nerve transfer and botulinum toxin injections to shoulder internal rotators	0	2	3.1

AMS: active movement scale.

*Excluded from analysis due to short follow-up.

**Table 3:** Results: patients for whom surgery was declined.

	Age (months)	Surgery details	Preoperative AMS for external rotation	Postoperative AMS for external rotation	Length of follow-up (months)
1	14.3	Declined	0	0	15.1
2	10.9	Declined	0	2	44.5
3	9.1	Declined	0	0	27.2

AMS: active movement scale.

## References

[1] Michelow BJ, Clarke HM, Curtis CG, Zuker RM, Seifu Y, Andrews DF (1994). The natural history of obstetrical brachial plexus palsy. *Plastic and Reconstructive Surgery*.

[2] Eng GD, Binder H, Getson P, O’Donnell R (1996). Obstetrical brachial plexus palsy (OBPP) outcome with conservative management. *Muscle Nerve*.

[3] Strömbeck C, Krumlinde-Sundholm L, Forssberg H (2000). Functional outcome at 5 years in children with obstetrical brachial plexus palsy with and without microsurgical reconstruction. *Developmental Medicine and Child Neurology*.

[4] Levine MG, Holroyde J, Woods JR, Siddiqi TA, Scott M, Miodovnik M (1984). Birth trauma: incidence and predisposing factors. *Obstetrics and Gynecology*.

[5] Hardy AE (1981). Birth injuries of the brachial plexus. Incidence and prognosis. *Journal of Bone and Joint Surgery B*.

[6] Gilbert A, Tubiana R (1993). Obstetrical brachial plexus palsy. *The Hand*.

[7] Gilbert A, Khouri N, Carlioz H (1980). Exploration chirurgicale du plexus brachial dans la paralysie obstétricale: constatations anatomiques chez 21 malades opérés. *Revue de Chirurgie Orthopédique et Réparatrice de l'Appareil Moteur*.

[8] Gilbert A, Tassin JL, Terzis JK (1987). Obstetrical palsy: a clinical, pathologic, and surgical review. *Microreconstruction of Nerve Injuries*.

[9] Fisher DM, Borschel GH, Curtis CG, Clarke HM (2007). Evaluation of elbow flexion as a predictor of outcome in obstetrical brachial plexus palsy. *Plastic and Reconstructive Surgery*.

[10] Clarke HM, Curtis CG (1995). An approach to obstetrical brachial plexus injuries. *Hand Clinics*.

[11] Borschel GH, Clarke HM (2009). Obstetrical brachial plexus palsy. *Plastic and Reconstructive Surgery*.

[12] Marcus JR, Clarke HM (2003). Management of obstetrical brachial plexus palsy evaluation, prognosis, and primary surgical treatment. *Clinics in Plastic Surgery*.

[13] Machin D, Campbell MJ, Fayers PM, Pinol APY (1997). *Sample Size Tables for Clinical Studies*.

[14] Zar JH (1984). *Biostatistical Analysis*.

[15] Waters PM (1999). Comparison of the natural history, the outcome of microsurgical repair, and the outcome of operative reconstruction in brachial plexus birth palsy. *Journal of Bone and Joint Surgery A*.

[16] Waters PM (2005). Update on management of pediatric brachial plexus palsy. *Journal of Pediatric Orthopaedics B*.

[17] Bahm J, Noaman H, Becker M (2005). The dorsal approach to the suprascapular nerve in neuromuscular reanimation for obstetric brachial plexus lesions. *Plastic and Reconstructive Surgery*.

[18] van Ouwerkerk WJR, Uitdehaag BMJ, Strijers RLM (2006). Accessory nerve to suprascapular nerve transfer to restore shoulder exorotation in otherwise spontaneously recovered obstetric brachial plexus lesions. *Neurosurgery*.

[19] Lapid O, Ho ES, Goia C, Clarke HM (2007). Evaluation of the sensory deficit after sural nerve harvesting in pediatric patients. *Plastic and Reconstructive Surgery*.

